# Switching Away from Utilitarianism: The Limited Role of Utility Calculations in Moral Judgment

**DOI:** 10.1371/journal.pone.0160084

**Published:** 2016-08-09

**Authors:** Mark Sheskin, Nicolas Baumard

**Affiliations:** Institut Jean-Nicod CNRS UMR 8129, Institut d’Etude de la Cognition, Ecole Normale Supérieure – PSL Research University, Paris, France; Brain and Spine Institute (ICM), FRANCE

## Abstract

Our moral motivations might include a drive towards maximizing overall welfare, consistent with an ethical theory called “utilitarianism.” However, people show non-utilitarian judgments in domains as diverse as healthcare decisions, income distributions, and penal laws. Rather than these being deviations from a fundamentally utilitarian psychology, we suggest that our moral judgments are generally non-utilitarian, even for cases that are typically seen as prototypically utilitarian. We show two separate deviations from utilitarianism in such cases: people do not think maximizing welfare is *required* (they think it is merely acceptable, in some circumstances), and people do not think that equal welfare tradeoffs are even acceptable. We end by discussing how utilitarian reasoning might play a restricted role within a non-utilitarian moral psychology.

## Introduction

Many moral decisions seem aimed at maximizing overall welfare (i.e., minimizing harms and maximizing benefits), consistent with an ethical theory called “utilitarianism.” A classic example from moral philosophy involves a runaway trolley that can be switched from a track where it will kill five people to an alternative track where it will kill only one person [1–2]. This case, and many variations on it, have been the focus of much recent work in moral psychology, with the majority of people judging that it is morally acceptable to maximize overall welfare, switching the trolley to the track with only one person (e.g., [3–7]).

Judgments in the idealized case of switching a trolley away from a larger group and towards a smaller group presumably reflect the motivations to increase others’ welfare that appear in many behavioral studies. Cross-cultural research has revealed that people across a wide variety of societies are willing to share some of a pool of money with a stranger (e.g., [8]). Developmental research has revealed that caring for others is early-emerging, with infants crying in response to others’ distress [9] and toddlers working to help others ([10], for a review see [11]). Comparative research with nonhuman animals has revealed that prosocial motivations can be found in a variety of other species, for example with chimpanzees helping another chimpanzee to access food ([12]; for a review see [13]). To be clear, a general prosocial motivation does not entail all of the specific requirements of utilitarianism (e.g., that it is immoral to act in a way that does not maximize utility), and indeed providing resources to others (as in many of the mentioned studies) can be consistent with either a utilitarian motivation or other motivations (e.g., for fairness).

Other judgments, across a wide range of domains, are clearly contrary to utilitarianism and motivations to increase general welfare, because they involve judgments *against* maximizing welfare. This is most notably the case when maximizing welfare (sometimes known as “efficiency”) conflicts with various conceptions of justice or fairness (for a review of justice theories, see [14]). For example, in making healthcare decisions, most people are unwilling to reduce cure rates for one group of ill people to increase cure rates for a larger group [15], even though increasing cure rates for the larger group would maximize welfare. Additional examples include that most people prefer income distributions based partially on equality rather than total income [16]; prefer retributive justice to deterrence, even though basing punishments on deterrence leads to lower crimes than basing punishments on retribution [17]; and condemn pushing one person off of a footbridge and in front of a trolley to save five people further down the tracks [5].

### Approaches to Moral Judgment Focused on Utilitarianism

Research has established very many influences on moral behavior besides utilitarianism, including constraints from reciprocity (e.g., [18–19]), respect for property (e.g., [20–21]), a desire for honesty (e.g., [22–23]), and, of course, competing motivations such as self-interest (e.g., [24–25]). However, utilitarian reasoning is often thought of as at least a core part of moral psychology, and it is sometimes used as the standard against which our moral judgments are measured, such that deviations from it must be described as biases or heuristics.

For example, Sunstein [26] argues that many of our moral judgments are based on heuristics that *typically* produce good output with great efficiency, but that are also susceptible to producing “absurd” judgments in a minority of cases. In line with this logic, it is generally good to condemn betrayal, but this leads people to prefer a car with no airbag to a car with an airbag that will save many lives but will also accidentally killing a small number of people (i.e., because the airbag is “betraying” its duty to protect life and indeed “murdering”). Thus, a rule-of-thumb that typically produces good consequences (e.g., “condemn betrayal”) leads people to judgments that are suboptimal in a minority of cases (e.g., disapproving of a technology that will lead to a net gain in lives saved).

Likewise, Greene [27] argues that genuine moral reasoning is typically based on utilitarianism, whereas deontological reasoning is often mere post-hoc rationalization for judgments led astray by other factors. Specifically, he argues that “deontological judgments tend to be driven by emotional responses, and that deontological philosophy, rather than being grounded in moral reasoning, is to a large extent an exercise in moral rationalization” (pg. 36). Greene places this in contrast with utilitarianism, which he argues, “arises from rather different psychological processes, ones that are more “cognitive,” and more likely to involve genuine moral reasoning” (pg. 36).

Furthermore, there are approaches to moral psychology that claim that *all* moral judgment is inherently about harm. Gray and colleagues [28] suggest that moral judgments follow a specific template of harm-based wrongdoing, in which a perception of immorality requires three components: (1) a wrongdoer who (2) causes a harm to (3) a victim. If any of these components appear to be missing, we automatically fill them in: “agentic dyadic completion” fills in an evil agent when a harm is caused, “causal dyadic completion” fills in a causal connection between an evil agent and a suffering victim, and “patientic dyadic completion” fills in a suffering victim in response to a bad action. For example, a person who perceives masturbation as immoral is likely to mistakenly attribute harm to *some* victim (e.g., “I believe you harm yourself, and so am motivated to believe masturbation leads to blindness”). In other words, perception of wrongdoing is a concomitant of a violation of utilitarianism (i.e., a net harm is occurring).

### Approaches to Moral Judgment that Include Utilitarianism

Other descriptions of the interplay between utilitarian and non-utilitarian judgments place the two on more equal footing. Many experiments investigate “dual-process morality” in which non-utilitarian judgments tend to be produced by quick cognitive mechanisms (sometimes characterized as “emotional”), and utilitarian judgments are produced by slower cognitive mechanisms (sometimes characterized as “rational”). Many of these approaches place an emphasis on the emotional judgments, an approach going back to David Hume [29] who claimed that “reason is, and ought only to be the slave of the passions.” More recently, Haidt [30] has characterized the subordination of reason to emotion as “emotional dog and its rational tail” (for a counterargument, see [31]; for a reply, see [32]). There is now a wide assortment of investigations and views about the interplay between reasoning and other factors in moral cognition (e.g., [6, 33–37]).

For example, Cushman and Greene [38] describe how moral dilemmas arise when distinct cognitive processes produce contrary judgments about a situation that do not allow for compromise. For example, a mother who is considering whether to smother her crying baby so that her group is not discovered by enemy soldiers might simultaneously recognize the utilitarian calculus that recommends smothering her baby, while still feeling the full force of non-utilitarian factors against killing her baby. There is no compromise between killing and not killing, and taking either action will violate one of the moral judgments, and so a moral dilemma results (see also [39]). The appearance of distinct moral motivations at the psychological level are mirrored by distinct neurological signatures (e.g., for equity and efficiency [40]).

Finally, the “moral foundations” approach advocated by Haidt and colleagues (e.g., [41–43]) suggests that a “harm domain” exists independent from other domains (e.g., a “fairness domain”), which might correspond to utilitarian judgments for promoting well-being separated from non-utilitarian judgments. The current taxonomy [41] includes six domains that are argued to be present in each individual’s moral judgments, though perhaps to different degrees (e.g., political liberals may focus disproportionately on harm and fairness, whereas political conservatives may tend towards an equal focus on all domains, [44]).

### Against Utilitarianism in Moral Judgment

In the current paper, we argue that even the case often taken as most prototypical of utilitarian reasoning (i.e., switching the tracks of the runaway trolley) shows two deviations from utilitarianism, suggesting that such moral judgments are *not* based on utilitarianism (e.g.,[45]). First, although people may judge that utility maximization is morally *acceptable* (in some cases), they do not think it is morally *required*. Second, people do not think equal utility tradeoffs (e.g., sacrificing one life for a different life) are even acceptable. The first point is established in Study 1 (Study 2 rules out an alternative explanation), and the second point is established in Study 3 (Study 4 rules out an alternative explanation).

Both of these points (*requiring* utility maximization and *allowing* any action that produces equally high utility as any other action) are standard features of utilitarianism. For example, in *Utilitarianism*, John Stuart Mill [46] describes the “Greatest Happiness Principle as “actions are right in proportion as they tend to promote happiness, wrong as they tend to produce the reverse of happiness.” This implies that actions that produce more happiness are more right, and that actions that produce equal happiness are equally right. Of course, different modifications to Mill’s original formulation may lead to different requirements, and it is possible to hold the view that actions with better consequences are required (the requirement we test in Study 1) while holding the view that tie breakers may occur for actions with equal utility, rather than either action being equally acceptable (the requirement we test in Study 3).

Importantly, previous studies have typically asked questions related to acceptability, rather than requirement. For example, Greene and colleagues [5] asked “Is it appropriate for you to hit the switch in order to avoid the deaths of the five workmen?”; Mikhail [7] asked “Is it permissible to push the button?”; Côté [4] provided a choice between “Yes, it is appropriate” and “No, it is not appropriate”; and Lombrozo [6] asked “Is it morally permissible for David to switch the train to the side track?” Importantly, Lombrozo [6] also asked a question that *is* related to requirement: “If David fails to switch the train to the side track, should he be punished?” It is possible (though not required) that participants would answer “yes” to this question if they thought switching was morally required and that people should be punished when they fail to do things that are morally required. However, the results for this question were not presented or analyzed in the paper.

Finally, our argument is consistent with a set of studies that were conducted by Royzman and colleagues independently of our own, and that were published as we were writing this paper ([37]; see also [47]). The studies by Royzman and colleagues show that people with higher scores on the Cognitive Reflection Test (indicating a tendency to inhibit immediate judgments and consider additional options) are less likely to support a strict utilitarian or a strict deontological response, and instead are more likely to support a “minimal” judgment in which utility-optimizing acts are permissible but not required.

## Study 1: Maximization Not Required

Study 1 investigated whether people think that maximizing utility is morally *required* for a straightforward case in which they typically judge that maximizing utility is morally *acceptable*. We randomly assigned 100 mTurk participants (60% male, mean age = 31.52 years, *SD* = 8.81) to either a Standard Switch case (“Do you think it is morally *acceptable* for John to switch the trolley to the other track?”) or a Required Switch case (“Do you think it is morally *required* for John to switch the trolley to the other track?”). The text for this, and all other studies, is in Appendix A.

In this study, and all subsequent studies, we used a sample size of 100, mTurk recruitment was limited to locations in the United States, and we did not exclude any participants from the analyses. This approach avoided increasing our false positive rate via “researcher degrees of freedom” [48]. Each study was run on a single day (ranging from October 2013 to January 2014 for the first four studies; the fifth study was added in May 2016), with the mTurk participants randomly assigned to condition by the Qualtrics online software that hosted our surveys.

Our research was conducted in compliance with the current French current laws regarding bioethics, information and privacy (Loi Informatique, Fichiers et Libertés), with current legislation about human subject research (which does not require IRB approval for research involving low risk methods such as computer-based data collection on cognitive judgments), and with the Helsinki declaration. Each participant provided written consent in the online survey before participating.

Each study was conducted using participants who had not participated in any of our previous studies, and each condition within a study was between-participants rather than within-participants. Although this means that we do not know how many individual participants would show each pattern of responses (e.g., endorsing an action as “acceptable, but not required”), this was a necessary design feature because previous research has shown that both non-experts and professional philosophers show strong order effects in questions such as these [49].

### Results

**Fig 1:**
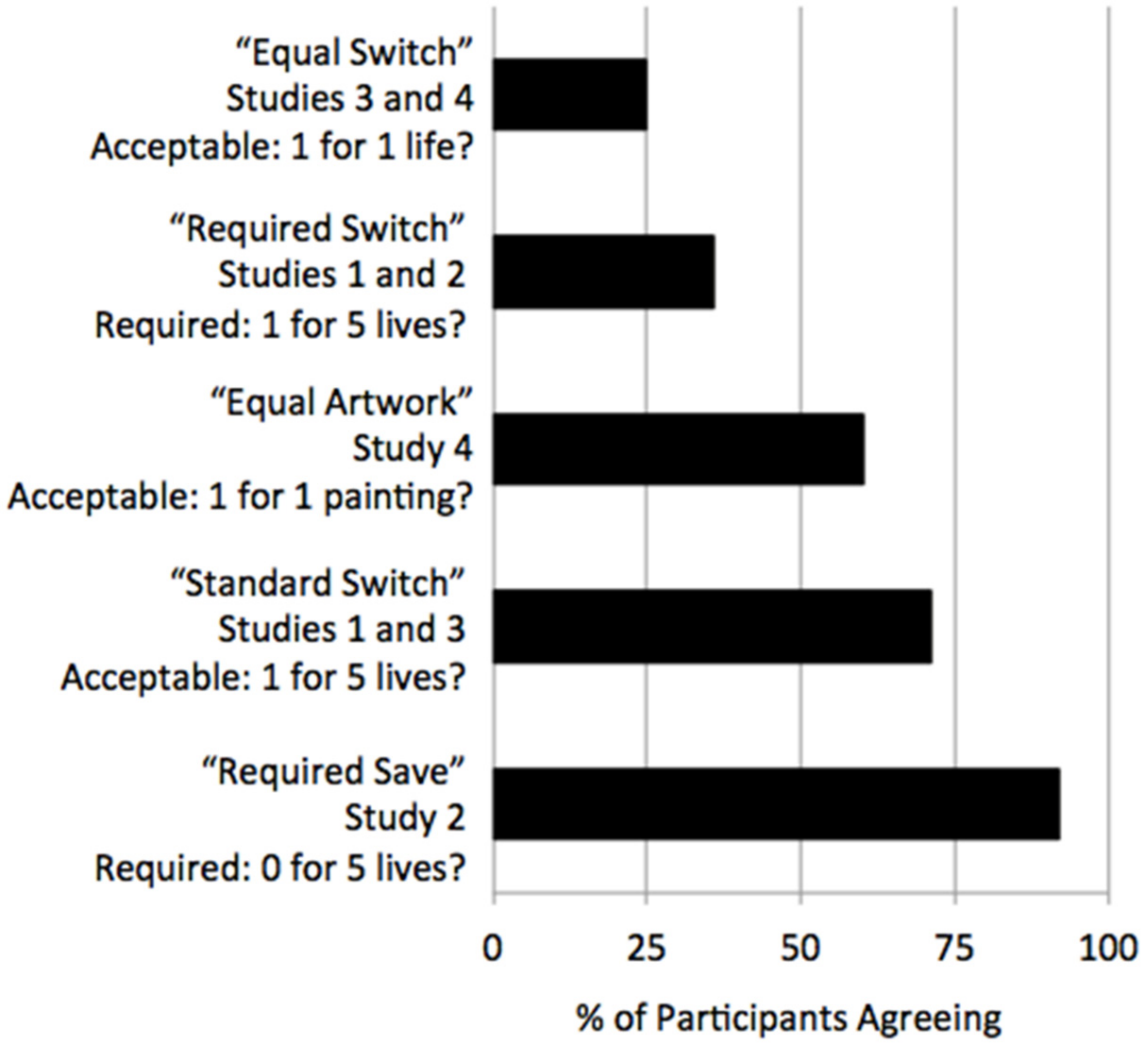
Summary of Studies 1 to 4. This bar chart reports the percent of participants agreeing with each of our cases, arranged in order of increasing agreement. Participants do not think it is acceptable to trade 1 life for 1 life, and they do not think it is required to trade 1 life for 5 lives. They are ambivalent about trading 1 painting for 1 painting. They do think it is acceptable to trade 1 life for 5 lives, and they do think it is required to trade 0 lives for 5 lives.

In the Standard Switch case, we replicated the standard result, in which the majority of participants judge it *acceptable* to switch the track (70% “acceptable,” binomial test, *p* = .003). However, in the Required Switch case, the majority of participants did not judge it *required* to switch the track (36% “required,” binomial test, *p* = .032). The difference between these conditions was significant (Fisher’s Exact, *p* = .001). A summary of the responses to these cases, as well as all the other cases presented throughout this paper, is presented in Fig 1.

### Discussion

We found that the majority of participants judge switching a runaway trolley from a set of tracks with 5 people to a set of tracks with 1 person to be “acceptable” but not “required.” This result is inconsistent with the demands of utilitarianism, and instead are consistent with Rozyman and colleagues [36], who found for a variety of other cases (e.g., smothering a baby to avoid detection by enemy soldiers) that a substantial percentage of participants will judge a utility-maximizing behavior as “permissible” but not “required.”

Importantly, participants who are moral nihilists (i.e., who do not think any actions are morally required) will answer for any action that performing the action is acceptable/permissible, but that the action is not required. Nihilistic judgments may be interesting in their own right, but they are distinct from more specific judgments that (although there *are* actions that are required) it is not required to maximize utility at the expense of a minority of individuals. In the studies by Royzman and colleagues [37], moral nihilism was evaluated by asking participants a follow-up question regarding whether “in this situation, there is no morally right or wrong answer.” We address the concern differently, by conducting a study using a minimal variation of our Required Switch case.

Specifically, we investigated whether people think it is required to save lives at no cost, using a new case that simply made the side track empty. In this “Required Save” case, the action maximizes welfare but does not require any cost. If the responses to the previous Required Switch case were the result of moral nihilism, then participants should show similar responses to the “Required Save” case. However, if the responses to the previous Required Switch case were the result of a specific judgment that maximizing welfare is not required when it harms a minority (but that welfare maximization *is* morally required when it is not costly), then participants should show the opposite pattern of results for the new Required Save case compared to the previous Required Switch case.

## Study 2: Some Actions Are Required

We randomly assigned 100 mTurk participants (50% male, mean age = 30.55 years, *SD* = 9.50) to either a Required Switch case (5 people on the main track and 1 person on the side track), or a Required Save case (5 people on the main track and 0 people on the side track).

### Results

We replicated our Study 1 result, in which people who received the Required Switch case did not judge it required to switch to a track with one person (36%, binomial test, *p* = .032). We found the opposite judgment in the Required Save case, in which participants did judge it required to switch to a track with zero people (92%, binomial test, *p* < .001). The difference between these conditions was significant (Fisher’s Exact, *p* < .001).

### Discussion

We found that the majority of participants think it is required to switch a runaway trolley from a set of tracks where it will kill five people to a set of tracks where no one will be harmed. More generally, most people *do* think that there are morally required actions (i.e., they are not moral relativists or nihilists); however, most people do *not* think that maximizing welfare at the expense of a minority is one of these required actions (even in the prototypical utilitarian example of the Switch case of the Trolley Dilemma).

## Study 3: Equal Trade-offs Not Acceptable

Study 3 investigated whether people think equal tradeoffs are acceptable. We randomly assigned 100 mTurk participants (58% male, mean age = 32.24 years, *SD* = 10.18) to either a Standard Switch case (5 people on the main track and 1 person on the side track) or an Equal Switch case (1 person on each track).

### Results

As in Study 1, the Standard Switch case replicated the standard result, in which participants judge it acceptable to switch the track to save five people (72%, binomial test, *p* = .001). However, in the Equal Switch case, they did not judge it acceptable to switch the track to save one person at the expense of a different person (28%, binomial test, *p* = .001). The difference between these conditions was significant (Fisher’s Exact, *p* < .001).

### Discussion

We found that the majority of people do not think it is acceptable to switch a trolley from a set of tracks where it will kill one person to a set of tracks where it will kill a different person. This result indicates a second deviation from utilitarianism: although people may say it is acceptable (though not required) to cause harm to bring about a greater benefit, they do not think it is even acceptable to cause harm to bring about an equal benefit.

This result might be specific evidence against equal tradeoffs in moral cases, or it might be more general evidence that people do not like to interfere with a status quo for no benefit. In other words, people may have judged trading one life for a different life as unacceptable because they think that *any* intervention in the world for no net gain is unacceptable. If so, then people’s anti-utilitarian judgments against welfare trade-offs would be the result of a more general status quo bias rather than a specific feature of morality. To investigate whether participants would judge a non-moral case with an equal tradeoff similarly to the Equal Switch Case, we introduced a new variation in which pieces of artwork replace the person on each track.

## Study 4: Some Equal Tradeoffs Are Acceptable

We randomly assigned 100 mTurk participants (58% male, mean age = 32.24 years, *SD* = 10.00) to either an Equal Switch case with 1 person on each track, or an Equal Artwork case with 1 painting on each track.

### Results

We replicated our novel Study 3 result, in which people who received the Equal Switch case did not judge it acceptable to switch the track for no net lives saved (22%, binomial test, *p* < .001). However, in the Equal Artwork case, participants did not show this aversion to switching the trolley away from one painting to another, though the result was not significant in the other direction (60%, binomial test, *p* = .101). The difference between the conditions was significant (Fisher’s Exact, *p* < .001).

### Discussion

People are ambivalent about whether it is acceptable to interfere with a non-moral status quo for no benefit. However, a significant majority of participants think it is not acceptable to interfere with a *moral* status quo for no benefit. Thus, people may have some level of a status quo bias (as indicated by the ambivalent results in the Equal Artwork case), but they have an additional aversion to equal tradeoffs with lives (as indicated by the significant result in the Equal Switch case, and the significant difference between the Equal Switch and Equal Artwork cases). Furthermore, these results are consistent with a range of additional cases tested by Kelman and Kreps [50], finding that participants are least willing to sacrifice for the greater good when lives are at stake, but are relatively more willing to sacrifice for the greater good for lesser harms such as injuries or property destruction.

## Study 5: Minimization is not Allowable

We randomly assigned 100 mTurk participants (62% male, mean age = 30.45 years, *SD* = 9.58) to two conditions that were the reverse of our first study: instead of a Standard Switch case (i.e., acceptable to switch from 5 to 1) and a Required Switch case (i.e., required to switch from 5 to 1), this study included a Reversed Standard Switch case (i.e., asking if it is acceptable to switch from 1 to 5) and a Reversed Required Switch case (i.e., asking if it is required to switch from 1 to 5). The text for these scenarios was identical to our first study, except for switching the numbers of people on each track.

Although nearly all theories about moral psychology have identical predictions for this study (i.e., that participants will think switching to kill more people is not required and not acceptable), we include this study to draw attention to the contrast between *doing* and *allowing* (alternatively described as “commission” vs. “omission”): whereas in our first study participants judged that it was *allowable* for a person to take no action (an omission) when taking no action led to five deaths rather than one, this last study establishes that people judge that it is *not* allowable for a person to take an action (commission) that leads to five deaths when the default is that one person dies. That is, the same outcome (five deaths) is allowable (though not required) when the result of omission, but not allowable when the result of commission. Thus, the *comparison* between this study and Study 1 demonstrates the influence of whether an outcome is achieved via an act vs. an omission.

### Results

Participants reported that it was not acceptable (82%, binomial test, *p* < .001) and not required (86%, binomial test, *p* < .001) to switch the trolley to kill more people.

### Discussion

Although in Study 1 participants reported (as is typical for the Standard Switch case) that it is acceptable to allow five people to die rather than to take an action that causes a single death, the participants in Study 5 reported (for the Reversed Standard Switch case) that it is not acceptable to take an action that causes five people to die rather than to allow a single death. These results highlight the doing/allowing (commission/omission) distinction, which is incompatible with a strict focus merely on outcomes (as in some forms of utilitarianism), though, as we will now describe in the General Discussion, these results are compatible with the two main approaches to moral psychology that we suggest may account for Studies 1 to 4.

## General Discussion

Moral psychology often places a large emphasis on utilitarian reasoning (e.g., [27]), or at least presents it as one of a small number of core parts of moral reasoning (e.g., [39]). In four studies, we show that even the “poster child” for utilitarian reasoning, the Switch Case of the Trolley Dilemma, shows two deviations from utilitarianism. First, people do not think it is required to switch a trolley to a track with fewer people (Study 1), even though they do think that some actions are morally required (Study 2). Second, people do not think it is acceptable to switch a trolley to a track with an equal number of people (Study 3), even though they are not so committed to the status quo in non-moral situations (Study 4). The non-utilitarian evaluation of these cases is emphasized in the comparison between our first and fifth studies, in which people indicate that it is acceptable to not switch a trolley from five people to one person (Study 1), but not acceptable to switch a trolley from one person to five people (Study 5): opposite judgments depending on whether the status quo requires an omission vs. a commission to lead to the superior outcome.

Moreover, even though Studies 1 through 4 are minimal variations on the switch case of the trolley dilemma, utilitarianism is in accordance with participants’ moral reasoning for only one of them. Importantly, this is the case in which no one is harmed (i.e., people think it is required to switch a trolley from a track where it will kill 5 people to a track where it will not kill anyone). This case clearly shows that people are willing to judge certain actions as morally required (i.e., they are not moral nihilists or relativists). However, as indicated by the other cases, avoiding harm is not considered in a utilitarian way, in which lesser harms *must* be committed to avoid greater harms, and harms may be committed to avoid equal harms.

Future research should investigate how our moral psychology takes harm into account. Here, we outline two alternatives: one possibility related to a moral psychology built around gaining a reputation for fairness, and a second possibility related to a moral psychology built around coordinating third-party condemnation.

The first possibility, that our moral psychology is centered on fairness (e.g., [51–53], suggests that we consider how to maximize welfare within the constraints of not violating fairness. This possibility is derived from recent work in evolutionary theory, which has suggested that our moral psychology is adapted for navigating a social environment in which people chose with whom to associate for mutualistic activities [45]. People who do not provide fair outcomes to others risk being shunned from future interactions in favor of fairer interaction partners. Thus, we only find it acceptable to maximize welfare when it is done in a mutually advantageous way that will not anger others. Specifically, we judge that each person should have equal access to welfare in any situation, taking into account variations in each person’s deservingness, based on relevant features such as their ex ante position or resources they have invested in the situation.

Applying this logic to the Trolley Dilemma, it may be acceptable to maximize numbers when several people are in an equally dangerous situation (such as walking along one or another set of trolley tracks in the Switch Case), but it is *not* acceptable to maximize numbers when doing so forces someone into a worse situation (such as violating the relative safety of a person who is in a secure spot on a footbridge in the Footbridge Case). This logic accounts not only for both of these standard cases, but also for the five new cases introduced in this paper. When lives can be saved at no cost, it is required to do so, because *all* of the individuals in the situation are benefiting equally. Otherwise, it is not required to maximize welfare, and may even be unacceptable if doing so inflicts an unfair cost on someone.

Applying this logic more broadly, this theory accounts for the fact that people allow welfare-maximization in some cases, but stop doing so when this would go against fairness. In other words, people allow actions to maximize the ends only when the means do not involve unfair actions such as actively killing someone (as in the prohibition on pushing in the Footbridge Case), acting unjustly (as in punishment decisions constrained by retributivist motivations), or producing inequality (as in economic decisions constrained by merit). Indeed, work by Tyler [54–55] suggests that people judge legal institutions as legitimate only to the extent that they see them as procedurally just. That is, differences in outcome are only allowable when they have been produced by a fair process.

Alternatively, a second possibility for how our moral psychology integrates harm is that we avoid causing explicit harm to others even when it leads to overall better outcomes because of features related to the coordination of third-party condemnation. As argued by DeScioli & Kurzban [56], moral cognition may be designed to respond to objective cues of wrongdoing that other bystanders can equally observe (i.e., not cues related to personal relationships, or subjective evaluations of situations), so that condemnation is only present when others are likely to share the costs of condemning. Likewise, moral cognition is geared towards avoiding acting so as to avoid being the target of coordinated condemnation of others. Thus, behaving in a way that causes recognizable harm to another should be done with great caution, even if it is likely to produce an better outcome overall.

Applying this logic to the Trolley Dilemma leads to similar results as the previously discussed fairness alternative: although it may be acceptable to maximize numbers when several people are in an equally dangerous situation (such as walking along one or another set of trolley tracks in the Switch Case), it is *not* acceptable to maximize numbers when doing so causes easily-identifiable harm to someone (such as violating the relative safety of a person who is in a secure spot on a footbridge in the Footbridge Case). Also like the fairness alternative, the condemnation alternative accounts not only for both standard trolley cases, but also for the four new cases introduced in this paper. When lives can be saved without causing harm, it is required to do so; otherwise, it is not required to maximize welfare, and may even be unacceptable if doing so inflicts harm on someone.

Both of these alternatives (fairness and third-party condemnation) are consistent with a well-established effect in moral psychology regarding “actions” vs. “omissions” (as in our Study 5). Specifically, people tend to judge an action that leads to a particular result more harshly than an omission (that is, a failure to act) that leads to the same result (e.g., [57–58]). In the trolley scenarios, failing to act to save more lives (e.g., the Standard Switch case in Study 1) is less likely to lead to a reputation for unfairness or to third-party condemnation) than acting to cause more death (e.g., the Reversed Standard Switch case in Study 5).

## Conclusion

We take it as instructive that much attention has been paid to why people find it *unacceptable* to fatally push the person in the Footbridge Case. For example, Greene and colleagues [59] suggest that the application of personal force plays a role in disallowing pushing the one person to save five others. Yet the judgment against killing the person on the footbridge is perfectly in line with the rest of moral judgments that condemn actions that inflict unfair costs on others (e.g. killing, stealing, etc.). The more surprising judgment is actually the Switch Case, in which people say it is acceptable to cause a death! In other words, what is in need of an explanation are not cases where people oppose harm to others, but cases where people allow it.

According to the fairness view, people will allow a death when they consider that killing one person is the solution that leads to mutual advantage, even taking fairness into account. For instance, people might consider that letting a terrorist group kill hostages (rather than paying the terrorists a ransom) is the best solution overall (this is in fact the official policy of most western countries). Here, people may consider that since paying a ransom increases the likelihood of hostage-taking and thus, *because people have equal chances of being taken hostage*, refusing to pay the ransom is the least bad solution from a the point of view of mutual advantage.

More generally, future research should investigate how harm is taken into account during moral judgments, given that harm is not evaluated in a utilitarian way. In the current paper, we have discussed two alternatives, one based on fairness and one based on coordinating third-party condemnation. For example, the previously-presented hostage situation is one in which harm is caused, but not to a specific identified individual. There is at least one version of the third-party condemnation alternative that would predict an aversion to causing harm to an individual even when that individual could not be identified in advance (i.e., you can be blamed for causing harm to Sally once she is the randomly-selected person who experiences the harm), whereas the fairness alternative does not predict such an aversion. Beyond research into judgments, research into the proximate mechanisms underlying moral judgment may differentiate between predictions of these two alternatives, and investigate additional questions (e.g., the extent to which explicit reasoning is implicated in moral judgments).

## Appendix A

Below are the 5 scenarios used across Studies 1 to 4, organized by increasing agreement (as in Fig 1). The titles were not visible to participants, and each participant selected one of the two statements at the end (i.e., “Yes…” or “No…”).

“Equal Switch” (Studies 3 and 4)

A runaway trolley is heading to a fork in the tracks, where it can go either to the right or to the left. On the right is one workman who will be killed if the trolley goes to the right. On the left is one workman who will be killed if the trolley goes to the left.

John is standing at a switch near the fork. He sees that the trolley is going to go to the right track with one person, and is trying to decide whether to throw the switch so the trolley instead goes to the left track with one person.

Do you think it is morally acceptable for John to throw the switch?

Yes, it is morally acceptable for John to throw the switch.
No, it is not morally acceptable for John to throw the switch.

“Required Switch” (Studies 1 and 2)

A runaway trolley is heading to a fork in the tracks, where it can go either to the right or to the left. On the right are five workmen who will be killed if the trolley goes to the right. On the left is one workman who will be killed if the trolley goes to the left.

John is standing at a switch near the fork. He sees that the trolley is going to go to the right track with five people, and is trying to decide whether to throw the switch so the trolley instead goes to the left track with one person.

Do you think it is morally required for John to throw the switch?

Yes, it is morally required for John to throw the switch.
No, it is not morally required for John to throw the switch.

“Equal Artwork” (Study 4)

A runaway trolley is heading to a fork in the tracks, where it can go either to the right or to the left. On the right is one painting, accidentally dropped during transit to a local museum, that will be destroyed if the trolley goes to the right. On the left is one painting, also accidentally dropped during transit to a local museum, that will be destroyed if the trolley goes to the left.

John is standing at a switch near the fork. He sees that the trolley is going to go to the right track with one painting, and is trying to decide whether to throw the switch so the trolley instead goes to the left track with one painting.

Do you think it is morally acceptable for John to throw the switch?

Yes, it is morally acceptable for John to throw the switch.
No, it is not morally acceptable for John to throw the switch.

“Standard Switch” (Studies 1 and 3)

A runaway trolley is heading to a fork in the tracks, where it can go either to the right or to the left. On the right are five workmen who will be killed if the trolley goes to the right. On the left is one workman who will be killed if the trolley goes to the left.

John is standing at a switch near the fork. He sees that the trolley is going to go to the right track with five people, and is trying to decide whether to throw the switch so the trolley instead goes to the left track with one person.

Do you think it is morally acceptable for John to throw the switch?

Yes, it is morally acceptable for John to throw the switch.
No, it is not morally acceptable for John to throw the switch.

“Required Save” (Study 2)

A runaway trolley is heading to a fork in the tracks, where it can go either to the right or to the left. On the right are five workmen who will be killed if the trolley goes to the right. There is no one on the left, and so no one will be killed if the trolley goes to the left.

John is standing at a switch near the fork. He sees that the trolley is going to go to the right track with five people, and is trying to decide whether to throw the switch so the trolley instead goes to the left track with nobody on it.

Do you think it is morally required for John to throw the switch?

Yes, it is morally required for John to throw the switch.
No, it is not morally required for John to throw the switch.
